# Identification and Verification of Diagnostic Biomarkers for Glomerular Injury in Diabetic Nephropathy Based on Machine Learning Algorithms

**DOI:** 10.3389/fendo.2022.876960

**Published:** 2022-05-19

**Authors:** Hongdong Han, Yanrong Chen, Hao Yang, Wei Cheng, Sijing Zhang, Yunting Liu, Qiuhong Liu, Dongfang Liu, Gangyi Yang, Ke Li

**Affiliations:** ^1^ Department of Endocrinology, the Second Affiliated Hospital, Chongqing Medical University, Chongqing, China; ^2^ Department of Endocrinology and Neurology, Jiulongpo People’s Hospital, Chongqing, China

**Keywords:** diabetic nephropathy, glomerular injury, biomarker, diagnostic model, machine learning algorithm

## Abstract

Diabetic nephropathy (DN) is regarded as the leading cause of end-stage renal disease worldwide and lacks novel therapeutic targets. To screen and verify special biomarkers for glomerular injury in patients with DN, fifteen datasets were retrieved from the Gene Expression Omnibus (GEO) database, correspondingly divided into training and testing cohorts and then merged. Using the limma package, 140 differentially expressed genes (DEGs) were screened out between 81 glomerular DN samples and 41 normal ones from the training cohort. With the help of the ConsensusClusterPlus and WGCNA packages, the 81 glomerular DN samples were distinctly divided into two subclusters, and two highly associated modules were identified. By using machine learning algorithms (LASSO, RF, and SVM-RFE) and the Venn diagram, two overlapping genes (PRKAR2B and TGFBI) were finally determined as potential biomarkers, which were further validated in external testing datasets and the HFD/STZ-induced mouse models. Based on the biomarkers, the diagnostic model was developed with reliable predictive ability for diabetic glomerular injury. Enrichment analyses indicated the apparent abnormal immune status in patients with DN, and the two biomarkers played an important role in the immune microenvironment. The identified biomarkers demonstrated a meaningful correlation between the immune cells’ infiltration and renal function. In conclusion, two robust genes were identified as diagnostic biomarkers and may serve as potential targets for therapeutics of DN, which were closely associated with multiple immune cells.

## Introduction

Diabetic nephropathy (DN) is a serious cause of end-stage renal disease, resulting in heavy economic and medical burdens. Tubulointerstitial lesions, glomerular basement membrane thickening, mesangial matrix accumulation, and nodular glomerulosclerosis are the basic pathological features of DN (1). The current treatment strategy is either to strengthen glucose control or to reduce glomerular intracapsular pressure to slow the progression of renal injury (2, 3). In fact, because of the individual heterogeneity of DN, not all patients can benefit from these drugs. Genome-wide expression profiles can be easily obtained from public databases, analyzed, and visualized on the R platform, thanks to the advancement and widespread application of bioinformatics analysis and high-throughput sequencing technology (4, 5). The changes in gene expression profiles involved in the initiation and progression of DN have been identified by high-throughput microarray technology (6).

According to the flow chart shown in **Supplementary Figure S1**, gene expression profiles of DN patients and normal samples were obtained and analyzed to identify the differentially expressed genes (DEGs). Highly associated modules were identified to determine the critical biomarkers, and a diagnostic model was developed based on the biomarkers. Moreover, enrichment analysis was performed to explore the potential mechanisms of the identified biomarkers in DN. It particularly illustrates the relationship between the biomarkers and immune cell infiltration.

## Materials and Methods

### Data Collection and Preprocessing

A total of fifteen human microarray datasets, namely GSE96804, GSE47183-GPL11670, GSE47183-GPL14663, GSE99339-GPL19109, GSE99339-GPL19184, GSE104948-GPL22945, GSE104948-GPL24120, GSE30122, GSE1009, GSE30528, GSE30529, GSE47184-GPL11670, GSE47184-GPL14663, GSE104954-GPL22945, and GSE104954-GPL24120, were downloaded from the Gene Expression Omnibus (GEO) database (http://www.ncbi.nlm.nih.gov/geo/). More details of the collected datasets are presented in **Table 1**. After eliminating the batch effects by the Surrogate Variable Analysis (SVA) algorithm (7), seven glomerular DN (GDN) datasets (GSE96804, GSE47183-GPL11670, GSE47183-GPL14663, GSE99339-GPL19109, GSE99339-GPL19184, GSE104948-GPL22945, GSE104948-GPL24120), three GDN datasets (GSE30122, GSE1009, GSE30528), and five tubulointerstitial DN (TDN) datasets (GSE30529, GSE47184-GPL11670, GSE47184-GPL14663, GSE104954-GPL22945, GSE104954-GPL24120) were merged, normalized, and utilized as the GDN training cohort, GDN testing cohort, and TDN testing cohort, respectively. The distribution patterns between DN and normal samples were visualized by principal component analysis (PCA).

**Table 1:** The essential information of included microarray datasets in this study.

GEO series	Normal	DN	Tissue	Data type
GSE96804	20	41	Glomerulus	Training
GSE47183-GPL11670	0	7	Glomerulus	Training
GSE47183-GPL14663	0	7	Glomerulus	Training
GSE99339-GPL19109	0	7	Glomerulus	Training
GSE99339-GPL19184	0	7	Glomerulus	Training
GSE104948-GPL22945	18	7	Glomerulus	Training
GSE104948-GPL24120	3	5	Glomerulus	Training
GSE30122	13	9	Glomerulus	Testing
GSE1009	3	3	Glomerulus	Testing
GSE30528	13	9	Glomerulus	Testing
GSE30529	12	10	Tubulointerstitium	Testing
GSE47184-GPL11670	0	7	Tubulointerstitium	Testing
GSE47184-GPL14663	4	11	Tubulointerstitium	Testing
GSE104954-GPL22945	18	7	Tubulointerstitium	Testing
GSE104954-GPL24120	3	10	Tubulointerstitium	Testing

### Identification of DEGs

DEGs between GDN and normal subjects in the GDN training cohort were detected by using the limma R package (8) with |log2 fold change (FC)|>1 and adjusted *p* < 0.05 as the cutoff threshold. Meanwhile, Gene Ontology (GO) enrichment analysis of DEGs was conducted using the clusterProfiler package. Gene Set Enrichment Analysis (GSEA) was also performed to investigate the significant differences in Kyoto Encyclopedia of Genes and Genomes (KEGG) pathways between GDN and normal samples, with the Molecular Signature Database (MSigDB)-derived gene sets “c2.cp.kegg.v7.4.symbols.gmt” selected as a reference. Enriched pathways with *p* < 0.05 and false discovery rate (FDR) <0.25 were considered statistically significant.

### Consensus Cluster Analysis

The ConsensusClusterPlus algorithm (9) was used to perform clustering analysis to identify potential subclusters of the GDN samples from the GDN training cohort. The maximum cumulative distribution function (CDF) index was selected as the optimal *k*-value. Meanwhile, principal component analysis (PCA) was employed to verify this classification based on gene expression patterns among different subgroups.

### Weighted Gene Coexpression Network Analysis

The Weighted Gene Coexpression Network Analysis (WGCNA) method (10) was applied to build potential modules related to different subclusters of the 81 GDN samples. After filtering abnormal samples and calculating the Pearson correlation coefficient, the correlation adjacency matrix was constructed. Highly associated modules were selected for subsequent analysis. Functional enrichments of the genes within given modules were performed to interpret the diverse biological effects based on the KEGG, GO, and Disease Ontology (DO) analyses using the ClusterProfiler, DOSE, and ggplot2 packages.

### Diagnostic Gene Screening and Diagnostic Model Construction

The Least Absolute Shrinkage and Selection Operator (LASSO) logistic regression (11), Support Vector Machine-Recursive Feature Elimination (SVM-RFE) (12), and Random Forest (RF) (13) algorithms were employed independently to screen the diagnostic genes from the selected modules. Ultimately, genes that overlapped among the three machine learning algorithms were regarded as diagnostic biomarkers. A receiver operating characteristic (ROC) curve was generated, and the area under the ROC curve (AUC) value was calculated to estimate the predictive utility of the identified biomarkers using the pROC package. The differential expression and predictive reliability of the biomarkers were further confirmed in the external testing cohorts. A diagnostic model was constructed using logistic regression analysis and visualized as a nomogram (14) to predict the glomerular injury in DN patients. The Concordance index (C-index), calibration curve, and decision curve analysis (DCA) were employed to visualize its discrimination performances. Besides, using the training datasets (**Table 1**), the expressions of the identified biomarkers were also explored in other chronic kidney diseases (CKD), including hypertensive nephropathy (HN) and systemic lupus erythematosus nephropathy (SLEN). Furthermore, based on the median expression level of each gene, 81 GDN samples from the GDN training dataset were divided into two groups (high- and low-expression group), and then Gene Set Variation Analysis (GSVA) was employed to clarify the enriched KEGG pathways with MSigDB gene sets “c2.cp.kegg.v7.4.symbols.gmt” used as a reference.

### Verification and Clinical Correlation Analysis of the Identified Biomarkers

The expression patterns of identified biomarkers were reconfirmed by the Nephroseq v5 online database (http://v5.nephroseq.org) (15). A correlation analysis between the biomarkers and renal function was also carried out.

### Evaluation of Immune Cell Infiltration

Based on the single-sample Gene-Set Enrichment Analysis (ssGSEA) method and the 29 gene sets of immune-related responses (16), the ssGSEA scores were quantified and designed to represent the activity and infiltrating fractions of immune cells and pathways in the GDN training cohort and the TDN testing cohort. The result of ssGSEA was shown as a heatmap. Furthermore, the cell-type identification by estimating relative subsets of RNA transcripts (CIBERSORT) algorithm (17) was performed to calculate the relative proportion of the infiltrating immune cells in each sample from the GDN training cohort and the TDN testing cohort. The abundances of infiltrating immune cells in DN patients and normal subjects were compared and visualized using the vioplot package. The differences in immune characteristics between the samples with low and high expression of the identified biomarkers were clarified. In the GDN training cohort, using the corrplot package, the correlations between the enrichment levels of infiltrating immune cells and the expressions of the diagnostic genes were also investigated.

### Animal Experiments

A total of 15 male C57BL/6 mice (8 weeks old; ~25 g) were purchased from the Chongqing Medical University Animal Experiment Center (Chongqing, China). Mice were randomly divided into normal groups (*n* = 5) and high-glucose-induced renal injury models (*n* = 10) and given access to a normal chow diet (NCD) or a high-fat diet (HFD) for 4 weeks. A mouse model of hyperglycemia was induced by an intraperitoneal injection of streptozotocin (STZ; Sigma-Aldrich, USA). The random blood glucose levels ≥16.7 mmol/L 72 h after the injection were considered a successful establishment (18). At the end of 8 weeks, five NCD mice and six HFD/STZ-induced mice were fasted overnight, blood and 24-h urine samples were collected, and then mice were sacrificed. The kidney was harvested for subsequent study. All animal experiments were carried out following the Guide for the Care and Use of Laboratory Animals, and the procedures were approved by the Research Ethical Committee of Chongqing Medical University.

Blood glucose levels were measured using the Roche Dynamic Blood Glucose Monitoring System (Roche, Mannheim, Germany) by blood sampling from the tail vein. Urine albumin, blood urea nitrogen (BUN), and serum creatinine (Scr) were detected using an automatic biochemical analyzer (Hitachi, Tokyo, Japan). The obtained renal tissues were fixed, embedded, and cut into slices. Subsequently, hematoxylin and eosin (H&E), Masson, Periodic Acid-Silver (PAS), Oil Red O staining, and immunofluorescence (IF) staining for the selected biomarkers were performed. The stained slices were visualized and pictured with a light or fluorescence microscopy (Olympus, Tokyo, Japan). According to the manufacturer’s instructions, the RT-qPCR was performed. The 2^−ΔΔCt^ method was used to quantify protein kinase cAMP-dependent regulatory type II beta (PRKAR2B) and transforming growth factor-beta-induced (TGFBI) expression with GAPDH as an internal control. The primer sequence is shown in **Supplementary Table S1**. A Western blot analysis was carried out. Primary antibodies against PRKAR2B (Santa Cruz, CA, USA) and antibodies against TGFBI (Abcam, Cambridge, UK) were used, respectively.

### Statistical Analysis

All statistical analysis was performed using the R software (version 3.6.3) or GraphPad Prism 8.0 (GraphPad Software, CA, USA). A Wilcoxon test was performed to compare immune cell infiltration and the identified biomarker expressions between normal subjects and DN patients. The logistic regression algorithm was used to develop the predictive model. A ROC curve was used to judge the diagnostic accuracy of selected biomarkers. Correlation analysis was realized by Pearson’s analysis. Moreover, an unpaired *t*-test was used to analyze the RT-qPCR, Western blot data, biochemical detection data, the differential expression levels of the two biomarkers from the Nephroseq v5 online database, and the differential expression levels of the two biomarkers in other CKD. If not specially indicated, *p* < 0.05 was defined as statistical significance.

## Results

### Identification of DEGs and Enrichment Analysis

**Figure 1:**
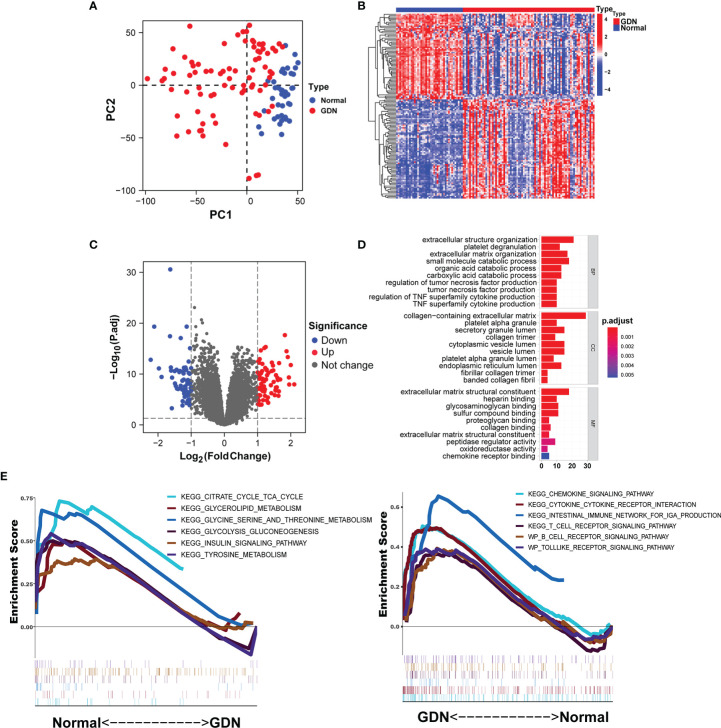
Identification of DEGs in the GDN training cohort. **(A)** The principal component analysis (PCA) for the samples. **(B, C)** Heatmap and the Volcano plot of the DEGs. **(D, E)** Six enriched signaling pathways in normal or DN samples.

There was a clearly pronounced discrimination between GDN and normal samples (**Figure 1A**). A total of 140 DEGs were identified including 75 upregulated and 65 downregulated genes, displayed in the Volcano plot and heatmap (**Figures 1B, C**). These DEGs were mainly involved in the biological processes associated with the extracellular structure organization and tumor necrosis factor production (*p* < 0.05, **Figure 1D**). The results of GSEA illustrated that metabolism-related pathways were enriched in the normal samples, while the immune-related signaling pathways were enriched in the GDN subjects (**Figure 1E**).

### Unsupervised Cluster Construction and Key Module Identification

**Figure 2:**
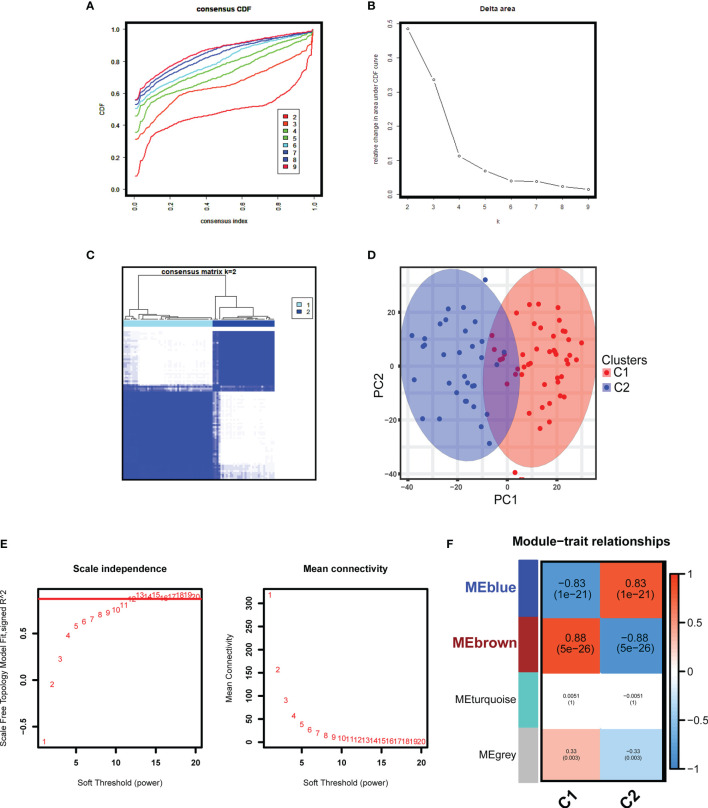
Unsupervised consensus clustering and WGCNA analyses in the GDN training cohort. **(A)** Cumulative distributive function (CDF) for *k* = 2 to 9. **(B)** Delta diagram showing the variations of the area under the consensus clustering CDF curve for *k* = 2 to 9. **(C)** Heatmap exhibiting the two clusters of DN samples with *k* = 2. **(D)** The principal component analysis (PCA) based on the results of consensus clustering analysis. **(E)** Analysis of the network topology for various soft-threshold powers. **(F)** Heatmap displaying the module-trait correlations.

With the batch effects stripped, the consensus clustering was performed based on the gene expression profiles of the merged 81 GDN samples in the GDN training cohort, and when *k* = 2, the classification was highly reliable and stable (**Figures 2A–C**). PCA confirmed that there was a distinct difference between the two subclusters (**Figure 2D**). GDN samples were divided into cluster 1 (C1, *N* = 48) and cluster 2 (C2, *N* = 33). With the soft-threshold power of *β* = 12 (scale-free *R* ^2^ = 0.906) set and the corresponding Pearson’s correlation coefficient calculated (**Figure 2E**), four modules were identified (**Figure 2F**). Brown and blue modules had the highest correlation with the subclusters, and therefore were selected as the associated modules for further analysis. The genes from the two selected modules were mainly responsible for extracellular structure organization and cytokine chemotaxis reactions (**Supplementary Figure S2A**). KEGG analysis indicated that they were significantly enriched in complement and coagulation cascades, PI3K-Akt signaling pathway and cytokine–cytokine receptor interaction (**Supplementary Figure S2B**). DO analysis revealed that the genes were mostly involved in urinary system disease, urinary system cancer, and lung disease (**Supplementary Figure S2C**).

### Diagnostic Biomarker Identification and Verification

**Figure 3:**
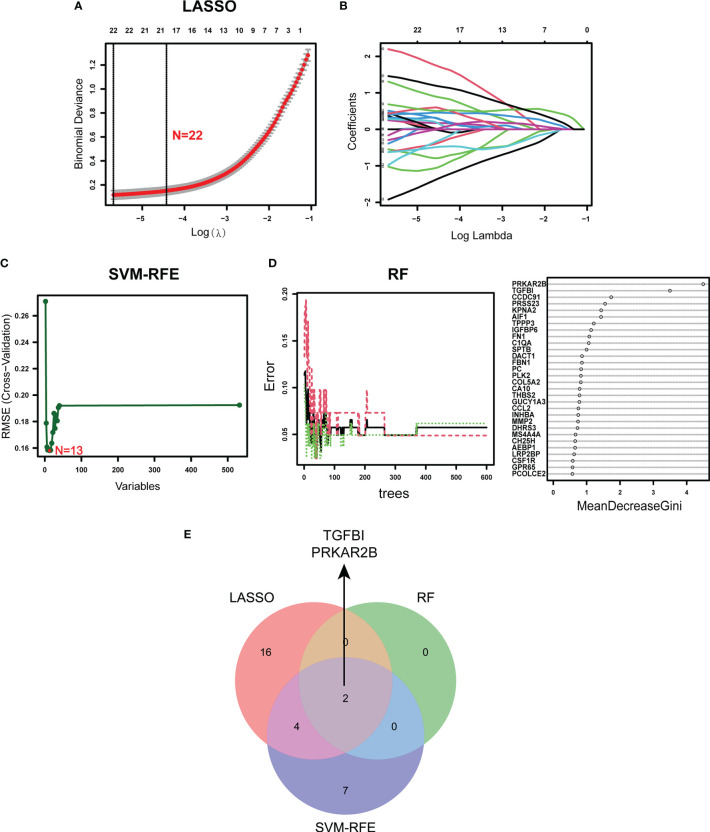
Identification of the diagnostic biomarkers from the selected modules. **(A, B)** LASSO regression analysis. **(C)** SVM-RFE algorithm. **(D)** RF algorithm. **(E)** Venn plot exhibiting the reliable biomarkers among LASSO, SVM-RFE, and RF.

**Figure 4:**
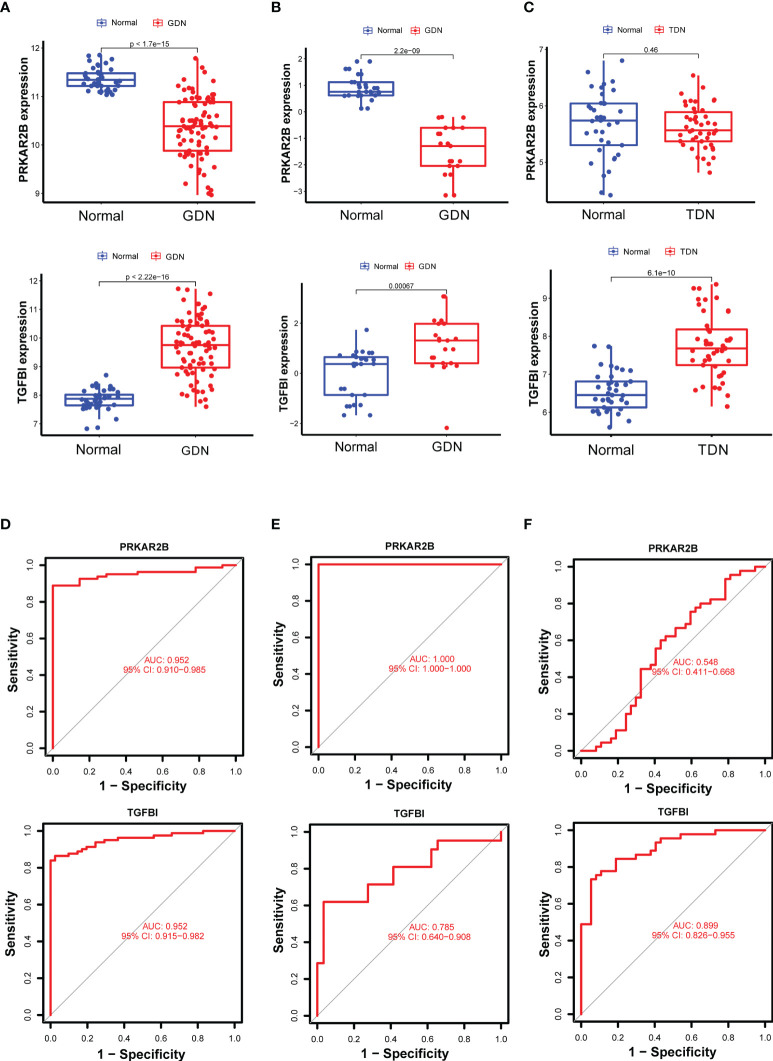
Verification of the identified biomarkers. **(A–C)** Box plots for the differential expression analysis in the GDN training cohort, GDN testing cohort, and TDN testing cohort, respectively. **(D–F)** ROC curves for evaluating the diagnostic ability in the GDN training cohort, GDN testing cohort, and TDN testing cohort, respectively. *p* < 0.05 was considered statistically significant.

Using the LASSO regression algorithm, 22 genes from the selected modules were identified as potential diagnostic biomarkers (**Figures 3A, B**). By SVM-RFE algorithm, 13 genes were extracted from these modules as candidate biomarkers (**Figure 3C**). Two diagnostic genes were identified by the RF algorithm (**Figure 3D**). Two genes (PRKAR2B and TGFBI) were then overlapped *via* a Venn diagram, and served as robust diagnostic biomarkers (**Figure 3E**). Compared with normal control, decreased PRKAR2B expression (*p* < 0.001) and increased TGFBI expression (*p* < 0.001) were observed in the glomerular samples from the GDN training cohort (**Figure 4A**). The results were validated in the GDN testing cohort, and the consistent gene expression patterns were obtained (**Figure 4B**). Interestingly, the expression of TGFBI was still significantly upregulated in tubulointerstitial samples from the TDN testing cohort (*p* < 0.001), while the expression of PRKAR2B had no significant change (**Figure 4C**). To estimate the predictive utility, the ROC curve was performed and found that the PRKAR2B and TGFBI illustrated a remarkably distinguishing efficiency with AUC values of 0.952 (95% CI: 0.910–0.985) and 0.952 (95% CI: 0.915–0.982) in the GDN training cohort, respectively (**Figure 4D**). Consistently, in the GDN testing cohort, the AUC value of PRKAR2B was 1.000 (95% CI: 1.000–1.000) and that of TGFBI was 0.785 (95% CI: 0.640–0.908) (**Figure 4E**). Unlike the low AUC value of PRKAR2B (0.548, 95% CI: 0.411–0.668), TGFBI still maintained a high AUC value of 0.899 (95% CI: 0.826–0.955) in the TDN testing cohort (**Figure 4F**). Furthermore, the similar expression patterns were also observed in HN and SLEN (**Supplementary Figure S3**).

### Establishment of Nomogram

**Figure 5:**
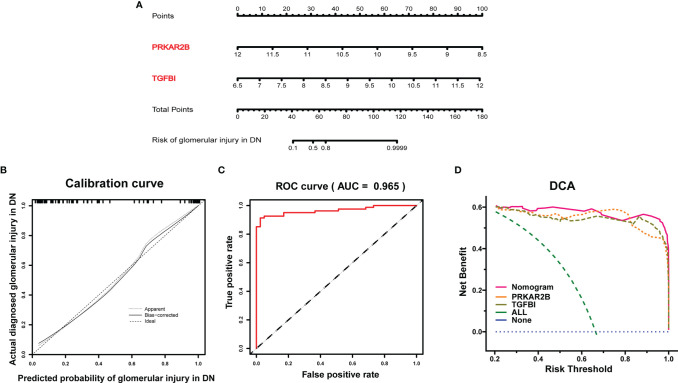
Establishment of the diagnostic model in the GDN training cohort. **(A)** Nomogram for the diagnostic model of glomerular injury. **(B)** Calibration curve. **(C)** ROC curves to evaluate the discrimination ability. **(D)** DCA for the diagnostic model.

Based on the expressions of PRKAR2B and TGFBI from the GDN training cohort, a diagnostic model was constructed by logistic regression and visualized as a nomogram (**Figure 5A**). The C-index of the diagnostic model was 0.976 with an appropriate calibration plot. Also, the model showed a high AUC value (0.965), confirming the excellent prediction performance (**Figures 5B, C**). Additionally, DCA curves indicated the combined nomogram model showed the highest efficacy in predicting glomerular damage in DN patients compared with other single biomarker models (**Figure 5D**).

### Expression Patterns and Clinical Correlation of the Biomarkers

**Figure 6:**
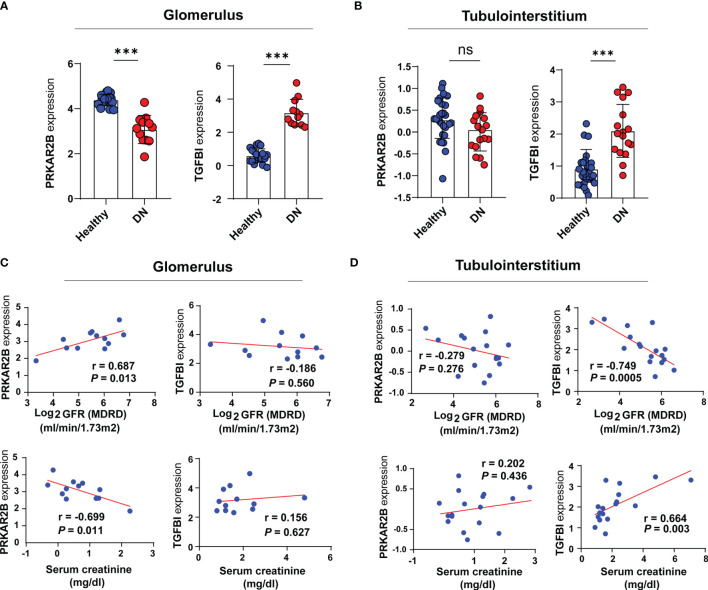
Verification of the two identified biomarkers. **(A, B)** The expression patterns of the identified biomarkers. **(C, D)** Correlation analysis between the expression of the biomarkers and renal function indexes. ^***^ *p* < 0.001 vs. healthy subjects. ns, not significant.

Based on the Nephroseq v5 online tool, the expression patterns of both PRKAR2B and TGFBI in the glomerular and tubulointerstitial tissues of DN patients were further confirmed (**Figures 6A, B**). When compared with normal subjects, PRKAR2B expression was downregulated in DN glomerular tissue but not in DN tubulointerstitial tissue. The TGFBI expression was upregulated in both glomerular and tubulointerstitial tissue of DN patients. Correlation analysis revealed that PRKAR2B expression in DN glomerular tissue was positively correlated with glomerular filtration rate (GFR) (*r* = 0.687, *p* = 0.013) and negatively correlated with Scr (*r* = −0.699, *p* = 0.011) (**Figure 6C**). The TGFBI expression in DN tubulointerstitial tissue was found to be negatively correlated with GFR (*r* = −0.749, *p* = 0.0005) and positively correlated with Scr (*r* = 0.664, *p* = 0.003) (**Figure 6D**). Curiously, the expression of TGFBI in DN glomerular tissue was not associated with GFR and Scr. It suggested that the biomarkers were related to renal function in patients with DN, whereas their roles may be different.

### Correlation Between the Two Biomarkers and Immune Cell Infiltration

**Figure 7:**
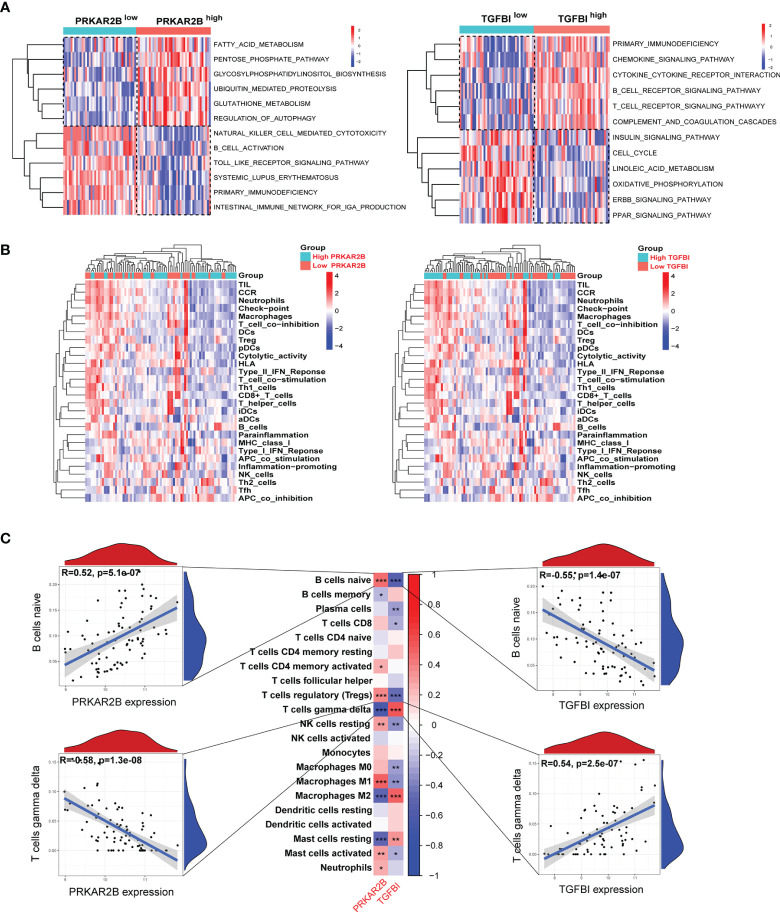
The association between the biomarkers and immune infiltration in the GDN samples. **(A)** Heatmap of metabolism and immune-related gene sets by GSVA. **(B)** Heatmap of the immune landscape by ssGSEA. **(C)** Heatmap of the correlations between the biomarkers and infiltrating immune cells. ^***^ *p* < 0.001; ^**^ *p* < 0.01; ^*^ *p* < 0.05.

The immune infiltration landscape in DN was obviously changed (**Supplementary Figure S4**). According to the GSVA results, the gene sets in the GDN samples with high PRKAR2B expression were markedly associated with multiple activated metabolism-related pathways and immune suppression biological functions, as well as the GDN samples with low expression of TGFBI (**Figure 7A**). Thus, given the roles of PRKAR2B and TGFBI in immune regulations, their effects on the immune cells’ infiltration and biological processes were also explored. A proliferation of neutrophils, regulatory T cells (Tregs), macrophages, and plasmacytoid dendritic cells (pDCs) were observed. In addition, the activities of check-point, tumor-infiltrating lymphocytes (TIL), chemokine C-C-Motif receptor (CCR), T-cell coinhibition, and type II interferon (IFN) response were markedly enhanced in the GDN subjects with low PRKAR2B expression or subjects with high TGFBI expression (**Figure 7B**). Correlation analysis revealed that the infiltration of naive B cells was most positively correlated with PRKAR2B and was most negatively correlated with TGFBI. However, the infiltration of gamma-delta T cells was most negatively correlated with PRKAR2B and was most positively correlated with TGFBI (*p* < 0.001). More details were exhibited in **Figure 7C**.

### Validation in Animal Models

**Figure 8:**
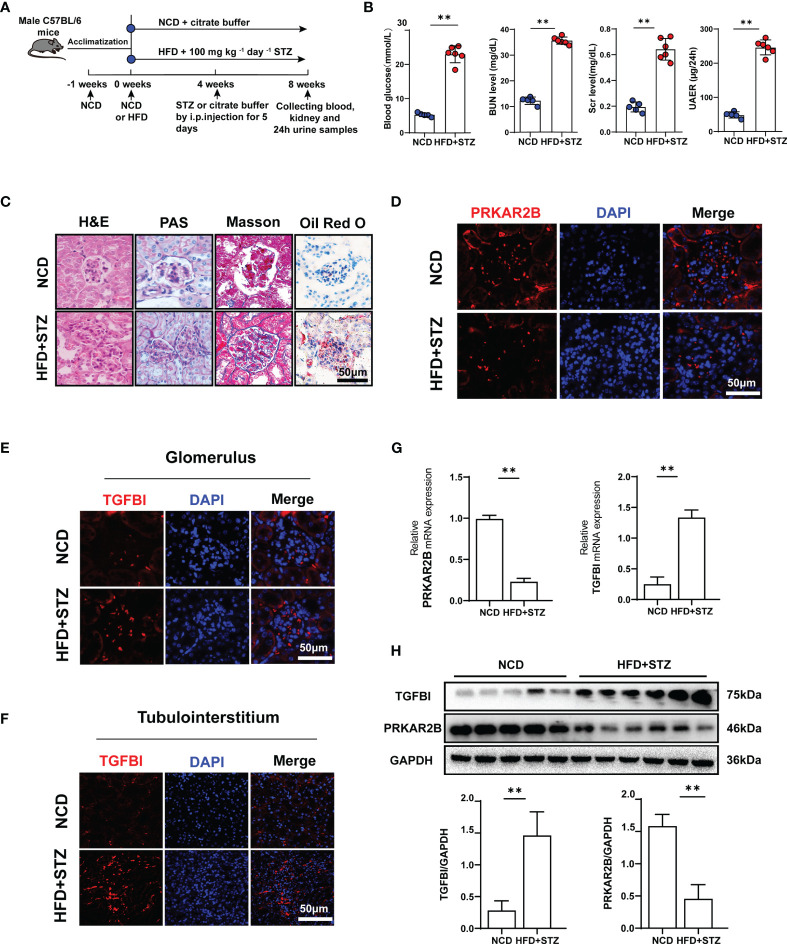
Verification in animal experiments. **(A)** The treatment protocol for mouse model. **(B)** Blood glucose, Scr, BUN, and 24 h proteinuria levels. **(C)** Renal pathological sections stained with H&E, PAS, Masson, and Oil Red O. **(D)** Immunofluorescence staining of PRKAR2B in renal glomerular tissue. **(E, F)** Immunofluorescence staining of TGFBI in glomerular and tubulointerstitial tissues. **(G)** The mRNA expression of PRKAR2B and TGFBI in kidney tissue (RT-qPCR). **(H)** The protein levels of PRKAR2B and TGFBI in kidney tissue (Western blot). ^**^ *p* < 0.01 vs. NCD mice. NCD, normal chow diet; HFD, high-fat diet; STZ, streptozotocin.

According to the treatment schedule (**Figure 8A**), four mice in the HFD+STZ group did not meet the established protocols and were excluded. The levels of blood glucose, Scr, BUN, and 24 h urinary protein were significantly elevated in the HFD/STZ-induced mice compared with the NCD mice (*p* < 0.01, **Figure 8B**). As shown in **Figure 8C**, glomerular hypertrophy, proliferation of glomerular mesangial cells, dilation of the mesangial matrix, and irregular thickening of the glomerular and tubular basement membrane were observed in the renal tissue of HFD/STZ-induced mouse model. Masson staining revealed the formations of renal blue-stained extracellular collagen, mostly in the glomerular tissue. Oil Red O staining showed the number of lipid droplets increased, and the lipid accumulation in the glomerulus was more obvious than that in the tubuleinterstitium. Thus, the HFD combined with high-glucose-induced renal injury model was considered successfully established. The downregulated PRKAR2B expression in glomerular tissue (**Figure 8D**) and the upregulated TGFBI expression in both glomerular and tubulointerstitial tissues of the mouse model were observed (**Figure 8E, F**). Moreover, the reduced PRKAR2B expression and increased TGFBI expression were also confirmed in the renal tissues of the mouse model by RT-qPCR and Western blot (*p* < 0.01, **Figure 8G, H**).

## Discussion

Diabetic nephropathy results from the interactions of multiple genes. However, its potential mechanisms remain unclear. Recently, a large number of studies have focused on the screening of related biomarkers. Wang et al. analyzed five DN-associated gene datasets and identified fibronectin 1 (FN1) and complement component 3 (C3) as the immune infiltration-related biomarkers for DN (19). Wang et al. revealed the different pathological abnormalities between glomerulus and kidney tubules in DN and indicated that the changes of key regulated genes in methylation status might contribute to the pathogenesis of DN (20). However, although many efforts have been made to explore novel targets for DN, the present knowledge seems to be insufficient. Potential biomarkers with high specificity and sensitivity are still urgently required.

PRKAR2B is a cAMP-dependent protein kinase (PKA) (21) regulatory subunit that is abundantly expressed in various tumor tissues (22). However, there are few studies on the role of PRKAR2B in the progression of DN. Our study identified that PRKAR2B, with an excellent diagnostic value (AUC >0.95), was downregulated in the glomerulus but there was no significant change in the tubulointerstitium. TGFBI is a secretory protein induced by TGF-β in various cells and can be detected in serum and urine (23, 24). It was demonstrated that TGFBI was involved in the fibrotic processes of chronic cyclosporine-induced nephropathy by affecting the synthesis and degradation of the extracellular matrix (25). In the present study, the expression of TGFBI was upregulated in both glomerular and tubulointerstitial tissues, and it was proved to have a reliable diagnostic ability for DN. It was reported that the expression of TGFBI was prominently increased in the kidneys of diabetic patients, whereas the concentration of TGFBI in urine was also raised (26). Elevated urinary TGFBI concentration has been shown to predict the prognosis of DN (27). This evidence enhanced the accessibility and feasibility of the clinical applications of TGFBI as a diagnostic marker. However, it is unclear why there was no significant correlation between TGFB expression in glomerular tissue and renal function indexes (such as GFR and Scr) in DN patients. Most notably, a novel diagnostic model combining the two biomarkers was developed with a high AUC value and favorable calibration, which exhibited excellent accuracy and reliability for estimating the glomerular damage in DN patients. Compared with any other single biomarker, the above model showed the highest efficacy for glomerular injury prediction in the GDN training cohort.

In this study, we found that the downregulated PRKAR2B expression in glomerular tissue may indicate the deterioration of kidney function in patients with DN, and so did the upregulated TGFBI in tubulointerstitial tissue. However, similar expression patterns of PRKAR2B and TGFBI were also found in patients with HN or SLEN, which suggested the differential expressions of PRKAR2B and TGFBI were not specific for DN but related to the renal injury.

It had been reported that extracellular matrix organization and extracellular matrix structural constituent lead to the accelerated deposition of extracellular matrix and renal fibrosis in DN (28). In this study, DEGs were demonstrated to be involved in this process in DN glomerular tissue. Multiple metabolism-related pathways were mainly enriched in normal samples, while immune inflammation pathways were mostly concentrated in GDN samples. It confirmed the notion that metabolic disorders and abnormal immune inflammation responses play a critical role in DN (29). Meanwhile, both PRKAR2B and TGFBI were disclosed to be involved in immune-related pathways and cell functions in the glomerular injury of DN. Moreover, both of them were associated with various immune cells such as naïve B cells, gamma delta T cells, Tregs, resting NK cells, resting mast cells, and macrophages. Previous studies reported that the deposition of macrophages, an important feature of DN, could be discovered in the kidney tissue of DN patients, indicating a decline in renal function (30). Mast cells were reported to participate in renal interstitial fibrosis, and the density of mast cells was related to serum creatinine levels in DN (31). It was reported that increased Tregs contributed to the improvement of DN and promoted the transplant tolerance to DN-induced renal allografts (32, 33). However, the roles of naïve B cells and gamma delta T cells in the pathological processes of DN have not been reported. Overall, the infiltrating immune cells are involved in the development and progression of DN. Improving abnormal immune status by targeting PRKAR2B and TGFBI may be a promising approach for the treatment of DN.

Some limitations need to be considered. First, different pathological stages of DN may affect the results of the study. Second, because of the potential heterogeneity from different annotation platforms and clinical covariates of samples, the batch effects cannot be completely eliminated among datasets. Third, the sample size may not be large enough. Finally, the present study was based on public data, so the biological functions of the two biomarkers need to be verified by further experiments.

In this study, using WGCNA, LASSO, SVM-RFE, and RF algorithms, PRKAR2B and TGFBI were identified as the potential biomarkers of DN. A diagnostic model combining PRKAR2B and TGFBI was established to evaluate the risk of diabetic glomerular injury with high sensitivity and accuracy. The potential association with infiltrating immune cells was also demonstrated, providing a fresh perspective on their roles in DN. Therefore, the findings may shed light on the management and treatment of patients with DN.

## Data Availability Statement

The original contributions presented in the study are included in the article/**Supplementary Material**. Further inquiries can be directed to the corresponding author.

## Ethics Statement

The animal study was reviewed and approved by the Research Ethical Committee of Chongqing Medical University.

## Author Contributions

HH, YC, and KL: conceptualization and methodology. HH, YC, HY, SZ, and WC: software and data curation. YL and QL: validation. DL and GY: reviewed and edited the manuscript. KL is the guarantor of this work and, as such, has full access to all the data in the study and takes responsibility for the integrity of the data and the accuracy of the data analysis. All authors listed have made a substantial, direct, and intellectual contribution to the work and approved it for publication.

## Funding

This study is supported by the National Natural Science Foundation of China (81671381), Natural Science Foundation of Chongqing (cstc2021jcyj-msxmX0169), Science and Health Joint Medical Research Project of Chongqing (2022GDRC018), High-Level Medical Reserved Personnel Train Project of Chongqing, and Kuanren Talents Program of the Second Affiliated Hospital of Chongqing Medical University.

## Conflict of Interest

The authors declare that the research was conducted in the absence of any commercial or financial relationships that could be construed as a potential conflict of interest.

## Publisher’s Note

All claims expressed in this article are solely those of the authors and do not necessarily represent those of their affiliated organizations, or those of the publisher, the editors and the reviewers. Any product that may be evaluated in this article, or claim that may be made by its manufacturer, is not guaranteed or endorsed by the publisher.
