# Supplementary material for: Identification and Verification of Diagnostic Biomarkers for Glomerular Injury in Diabetic Nephropathy Based on Machine Learning Algorithms
Source: Front Endocrinol (Lausanne). 2022 May 19;13:876960. doi: 10.3389/fendo.2022.876960 (PMC9162431; doi:10.3389/fendo.2022.876960)
Supplement: Supplementary file 1 [file DataSheet_1.pdf]

Figure S1. Flow chart

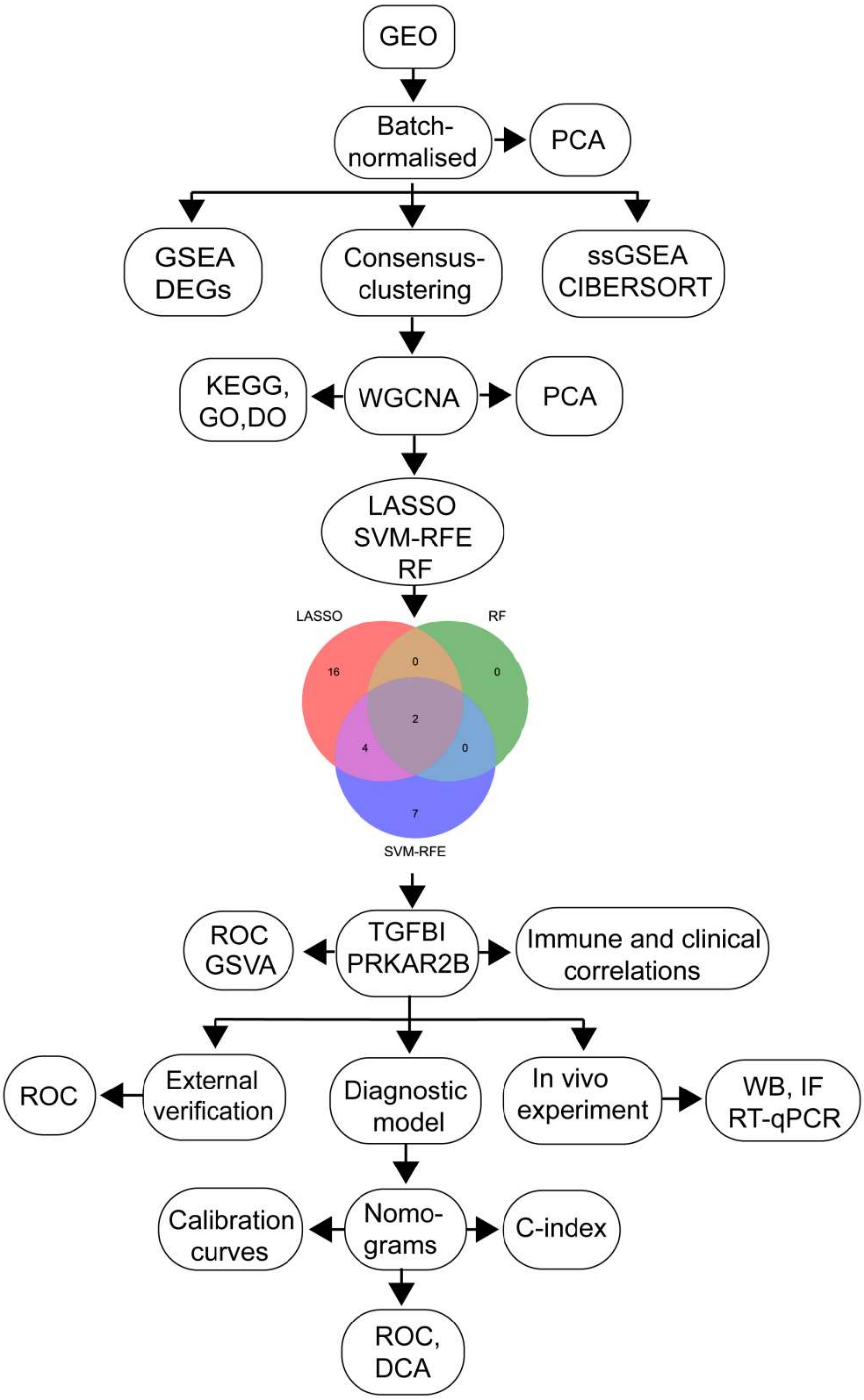

Figure S2. Functional analyses involving in (A) GO, (B) KEGG pathways and (C) DO of the genes in the blue and brown modules.

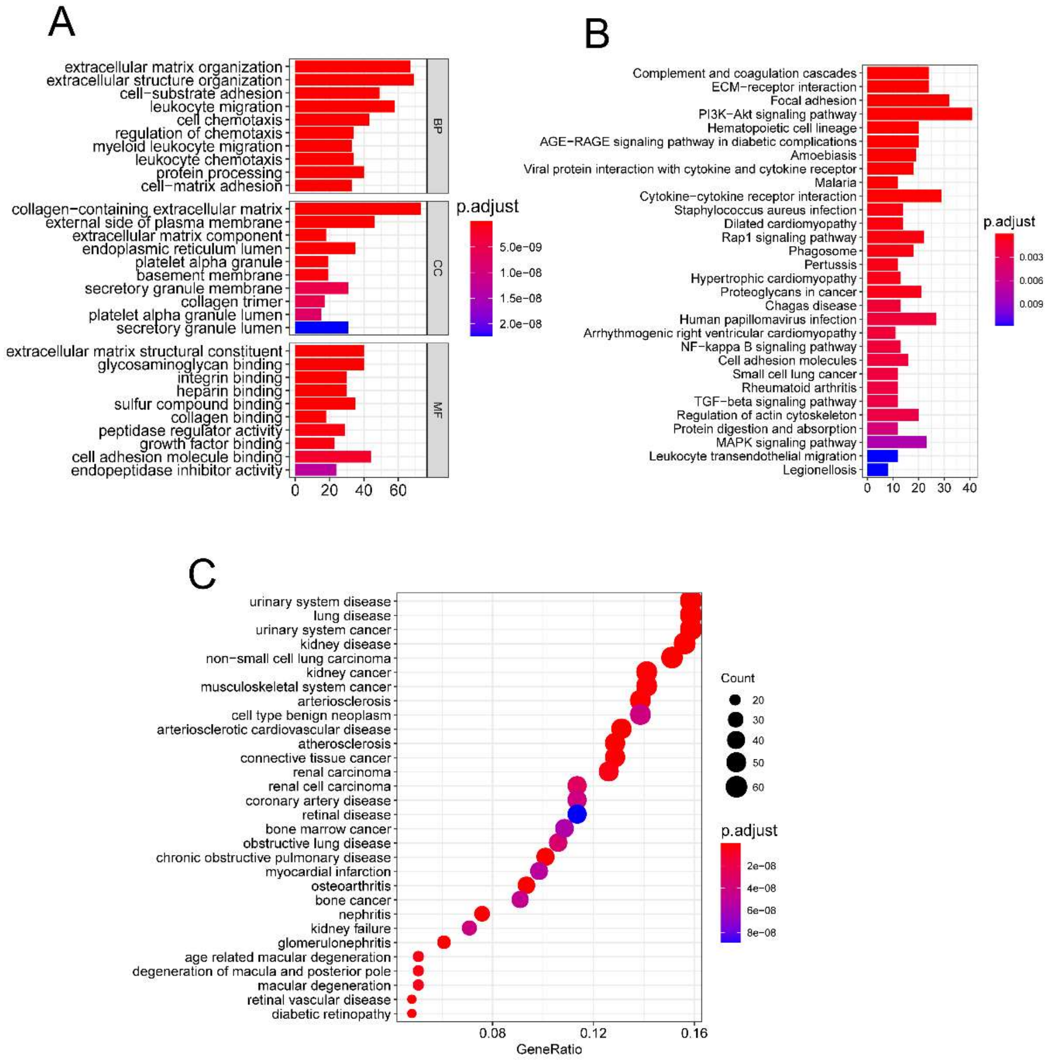

Figure S3. Verification of the two biomarkers in patients with (A) HN and (B) SLEN from the train datasets. ***$P<0.001$ *vs.* the normal subjects. HN, hypertensive nephropathy; SLEN, systemic lupus erythematosus nephropathy.

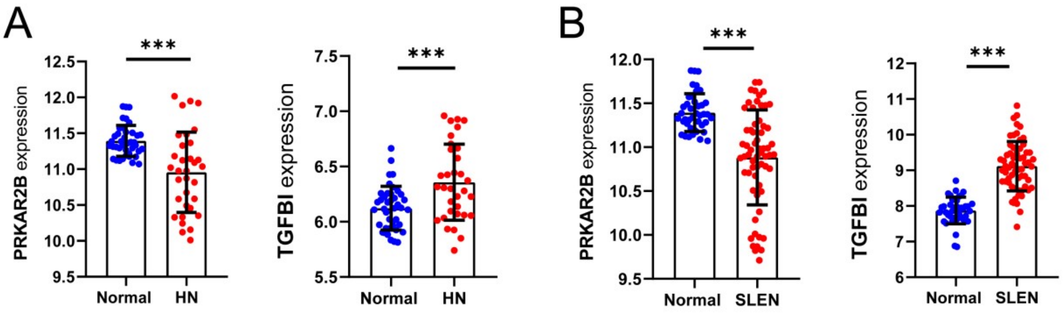

Figure S4. Immune cell infiltration profiles between normal and DN samples (**A**-**B**) Heatmap of the infiltrating immune cells and immune functional features. (**C**-**D**) Violin plot of the proportion of immune cell types in the GDN train cohort and the TDN test cohort.

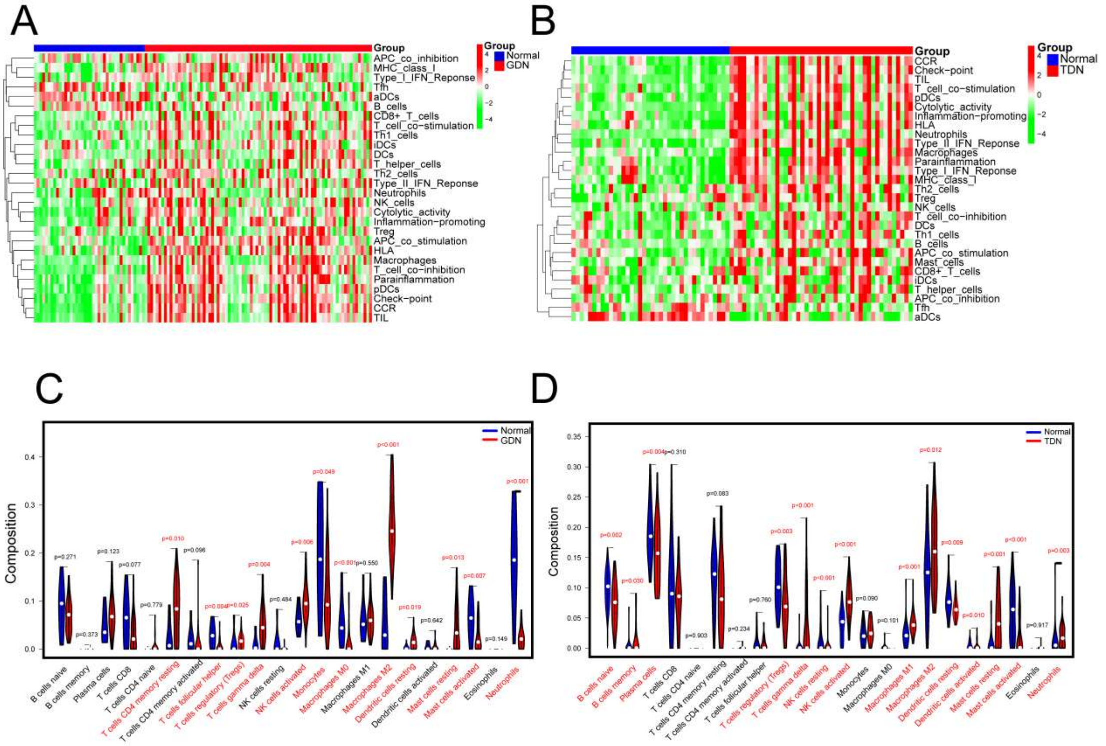

Table S1. RT-qPCR primers.

Gene name	Forward primer	Reverse primer
PRKAR2B	GTTCAACGCTCCAGTTATAAACCG	TTATCCTGGACTCTGCATCGTCT
TGFBI	GTTCACCATGGACCGGATGT	GGCCACCAGCATGCTAAAAC
GAPDH	TCTCTGCTCCTCCCTGTTC	ACACCGACCTTCACCATCT
